# Pathways from non-communicable diseases to depression among older adults in India

**DOI:** 10.1007/s00127-026-03146-9

**Published:** 2026-06-15

**Authors:** Kanchan Yadav, Dechenla Tshering Bhutia

**Affiliations:** Department of Community Medicine, Sikkim Manipal Institute of Medical Sciences (SMIMS), Sikkim Manipal University (SMU), Tadong, Gangtok, Sikkim, 737102 India

**Keywords:** Depression, NCDs, Structural equation modeling, Sustainable development, Latent variable, India

## Abstract

**Background:**

Depression co-occurring with non-communicable diseases is a major public health concern among ageing population, particularly in low and middle-income countries like India. But, the complex hidden patterns, predictors and interrelationships among functional health, lifestyle behaviours and NCDs burden in shaping depressive symptoms remain insufficiently explored.

**Methods:**

The present study utilized data from the Longitudinal Ageing Study in India Wave-1 (2017–2018), comprising 59,298 individuals aged 45 years and above. Exploratory and confirmatory factor analyses were conducted to derive latent constructs of functional limitations, lifestyle behaviours and NCDs burden. Structural equation modeling was used to examine direct and indirect associations with depressive symptoms and ordinal regression modeling was applied to assess important predictors of depression severity.

**Results:**

Approximately 40% of participants reported some level of depressive symptoms. Three latent domains were identified, representing functional limitations, lifestyle behaviours and NCDs burden. The SEM showed acceptable fit (CFI = 0.923, RMSEA = 0.062). Functional limitations exhibited the strongest positive association with depressive symptoms (β = 2.052, *p* < 0.001), followed by NCDs burden and lifestyle behaviours. Ordinal regression modeling results were consistent with functional impairment and poor self-rated health emerging as key predictors of higher depression severity.

**Conclusion:**

The findings suggest that functional health plays a central role in the relationship between chronic disease burden, lifestyle factors and depressive symptoms. Interventions aimed at maintaining functional ability, alongside integrated management of NCDs and promotion of healthy behaviours, may help improve mental health outcomes among ageing population.

**Supplementary Information:**

The online version contains supplementary material available at https://doi.org/10.1007/s00127-026-03146-9.

## Introduction

 Inevitable ageing, considered as continuous increasing phase of life-course often overlooked and underreported, is a major demographic shift globally and particularly in India, where the proportion of older adults is steadily increasing. As older individuals are growing, it creates both opportunities and challenges for public health systems. Nowhere is this transformation more evident than in India, which is projected to host over 319 million people aged 60 years and above by 2050, nearly one-fifth of its population [1, 2]. This demographic shift has subtle implications for sustainability in healthcare organizations, social support and economic productivity [3, 4]. To ensure that older adults can live healthy, meaningful and socially connected lives, which is central to the principles of sustainable development, and emphasize the interdependence of physical, psychological and social well-being across the life course [5, 6]. Among these trials and hurdles, the mental health of older adults has emerged as a critical concern. Particularly, depression in later life carry substantial implications for quality of life, functional independence and physical health trajectories.

Depression is one of the most common and often undertreated mental disorders among older age-groups and remains a leading contributor to disability worldwide. The WHO reports that depression is the single largest contributor to global disability, accounting for substantial amount of years lived with disability [7, 8]. Although, in India, national‐level estimates based on self‐reported diagnosed depression among older adults may appear relatively low, only 0.6% of older adults aged ≥ 60 years, reported diagnosed depression in large survey [9, 10], systematic reviews and meta‐analyses suggest a considerably higher prevalence of depressive symptoms. A previous meta‐analysis of 51 studies from 16 Indian states found a pooled prevalence of 34.4% of depression among individuals aged ≥ 60 years [8]. Another study for older adults aged 45+ reported an age‐standardised prevalence of 5.7%, though this estimate likely underrepresents milder forms of depression due to restrictive diagnostic criteria [10]. These contradictory estimates highlight the heterogeneity in measurement approaches, screening tools and definitions of depression. However, appreciable proportions of older adults in India experience depressive symptoms.

In later life, depression is not only prevalent but also highly consequential as it is associated with decreased physical, cognitive and social functioning, as well as poorer prognosis of other comorbid conditions [8, 11]. Additionally, older adults with depressive symptoms are more likely to experience higher healthcare use, greater disability and reduced quality of life. Researches across both high-income and low and middle‐income countries have advanced and enlighten our understanding of how physical health, functional capacity and behavioural risk factors interact to influence mental health in older age. The presence of multiple chronic non‐communicable diseases (NCDs) such as diabetes, cardiovascular disease, chronic respiratory disease and cancer, has been repeatedly shown to increase the risk of depression among older adults, partly by undermining physical functioning, autonomy and psychosocial well-being [12, 13]. The bidirectional relationship between mental health and NCDs represents a growing sustainability challenge, untreated and ignored depression can worsen adherence to medication or lifestyle modification, while chronic physical illness can exacerbate depressive symptoms [14]. Addressing this interaction is therefore not only a clinical imperative but also a fundamental need for sustainable development, as achieving health targets within the Sustainable Development Goals (SDG 3) depends on integrating physical and mental well-being.

Functional limitations captured by Activities of Daily Living (ADL), Instrumental ADL (IADL) and mobility restrictions mediate this association, serving as proximal determinants of depressive symptoms. Previous studies have shown that limitations in ADL and IADL were significantly associated with higher odds of depression among older adults in India [10, 13]. Similarly, intervention studies highlight that effective management of depression in later life should also aim to restore functional capacity rather than only target symptomatic relief [11]. In parallel, lifestyle behaviours including smoking, alcohol consumption, physical inactivity and social inactivity have been implicated in late-life depressive symptomatology. Although the magnitude of effect may be smaller compared to physical or functional health burdens, these behaviours are modifiable and therefore of considerable public health relevance. For instance, meta‐analyses indicate smoking elevates odds of depression (OR ~ 1.16) and physical activity is associated with modest reductions in depressive symptoms (OR ~ 0.94) among older adults [15]. Social participation and engagement also function as important behavioural and psychosocial protective factors against depression, because their absence increases the risk of depressive symptoms by reinforcing social isolation and functional decline [13].

According to recent meta-analysis, India is experiencing rapid demographic transition by 2050, the share of population aged 60 years and above is projected to reach ~ 19% (from ~ 8.6% in 2011) [8]. This demographic shift places additional burden on health systems that are already confronting rising prevalence of NCDs, resource constraints and persistent inequities in access to care across rural/urban and socio-economic strata. Previous studies in India highlight that older adults who are female, reside in rural areas, have lower levels of education, were widowed or live alone or report poor self‐rated health are at an elevated risk of depression [10, 13]. Recent evidence from India has begun to examine the relationship linking ageing, multimorbidity and well-being outcomes. A recent study [16] using LASI data demonstrated that functional health mediates the relationship between multimorbidity and quality of life among older adults, highlighting the central role of physical activity in shaping broader health outcomes. Despite the growing body of evidences, important key gaps remain. First, many existing studies focus primarily on prevalence and associated factors rather than modeling the interrelationships among NCDs burden, physical functioning, lifestyle behaviours and depressive outcomes. Second, very few studies in India have employed latent‐variable modelling or structural equation modeling (SEM) techniques to disentangle direct and indirect pathways linking these domains to depression in older adults. Third, while functional limitations are widely recognized as a risk factor for depression, the relative contributions of lifestyle behaviours and NCDs burden, as well as their mediating inter‐relationships remain underexplored in large, nationally representative samples of older adults in India.

According to this scenario, the present study aims to fill these gaps by using the large-scale LASI Wave 1 data to examine how multiple dimensions of health and behaviour namely functional limitations, lifestyle behaviours and NCDs burden are interrelated and contribute to depressive symptoms. Specifically, the study seeks to describe the socio-demographic, health-related, behavioural and functional profile of older adults in India, as well as extract latent constructs representing functional limitations, lifestyle behaviours and NCDs burden through exploratory and confirmatory factor analyses. Thereafter, estimate direct and indirect pathways linking these latent constructs to depressive symptomatology through structural equation modeling and also assess the relative magnitude of these pathways and their implications for prevention and intervention among older adults in India.

**Fig. 1:**
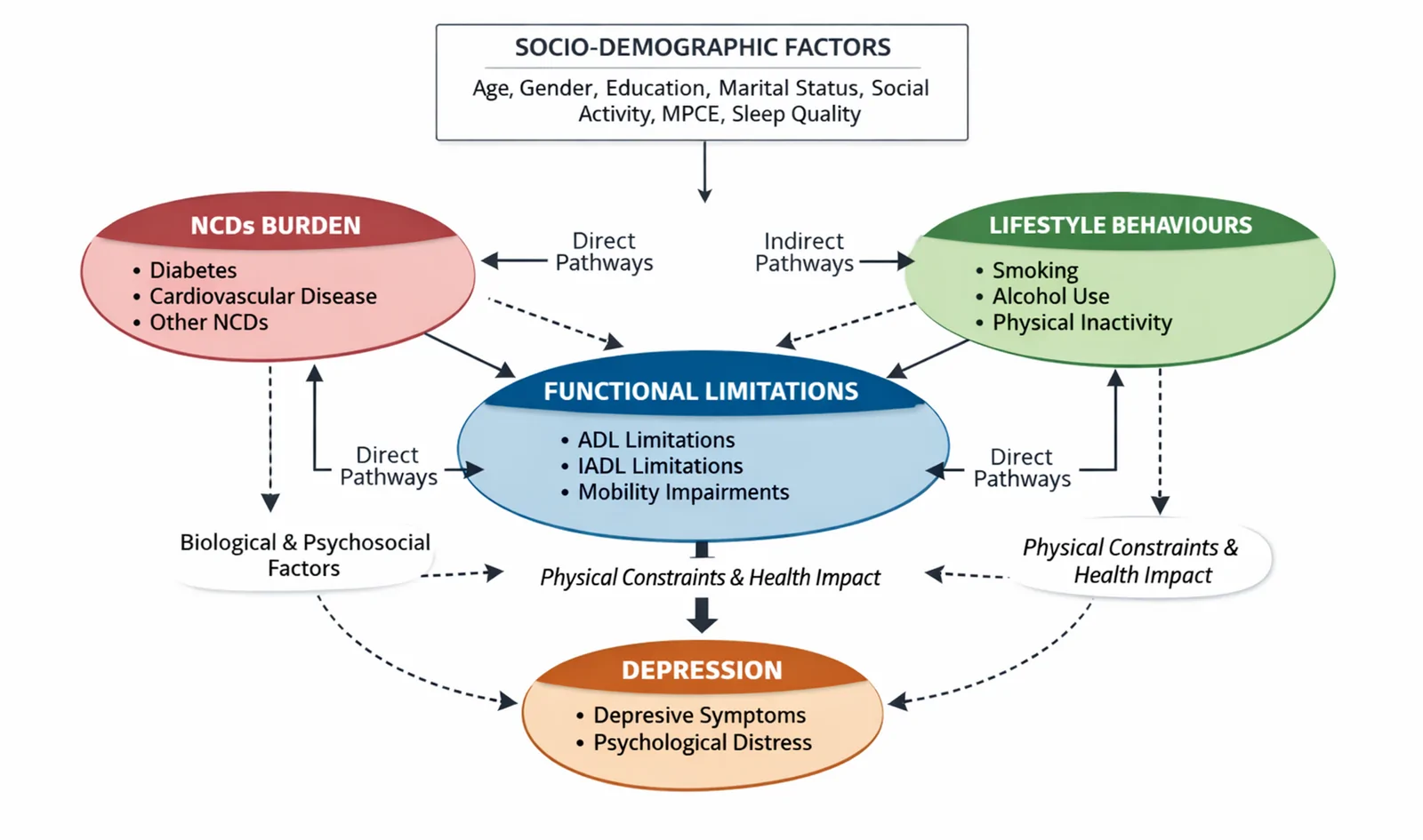
Conceptual Framework of Hypothesized Pathways Linking NCDs, Lifestyle Behaviours, Functional Limitations and Depression

For providing a stronger theoretical foundation for the analytical framework, the present study conceptualizes depression in later life as a multidimensional outcome shaped by interrelated physical, behavioural and clinical domains. Drawing on the disablement process model proposed by Verbrugge and Jette [17] and the biopsychosocial framework introduced by George L. Engel [18], the study groups observed variables into three latent constructs namely functional disability, lifestyle behavioural changes and chronic diseases burden. Functional disability or limitations, captured through ADL, IADL and mobility restrictions, represent proximal determinants of well-being, reflecting loss of independence and reduced capacity to perform everyday activities, which are strongly associated with depressive symptoms in older age. Lifestyle behavioural changes, including smoking, alcohol consumption, physical inactivity and social inactivity, are conceptualized as modifiable behavioural risk factors that influence both physical health and psychological outcomes. The chronic diseases burden domain, comprising of chronic conditions such as diabetes and cardiovascular disease, reflects underlying physiological health status that contributes directly to depression and indirectly through pathways such as functional decline and reduced well-being of life. This tripartite conceptualization is supported by prior epidemiological and gerontological research demonstrating that chronic disease, functional impairment and lifestyle behavioural risks jointly shape mental health outcomes in ageing population [17, 19–21]. Thus, the specification of these latent constructs is theoretically grounded and subsequently validated through empirical factor analysis, forming the basis for the structural equation modeling framework employed in this study. A conceptual framework presented herewith the hypothesized relationships among NCDs burden, lifestyle behaviours, highlighting the mediating role of functional limitations and depression (showed in Fig. 1).

Despite medical or clinical advancements, the coexistence of NCDs and depression continues to pose a significant public health challenge. By adopting a latent-variable SEM framework, complemented by ordinal regression modeling, this study contributes to theory by clarifying the proximal and distal determinants of depressive symptoms in older age and to practice by identifying prioritised domains for screening and intervention within the Indian ageing context. The findings of the present study are particularly relevant for policymakers, geriatric healthcare providers and mental health professionals seeking evidence‐based strategies to reduce the burden of depression among ageing population of developing countries like India.

## Materials and methods

### Data source, sampling procedure and sample size

Data from the first phase of the Longitudinal Ageing Study in India (LASI), carried out between 2017 and 2018, was used for the analysis. The research partnership for LASI included the International Institute for Population Sciences (IIPS), the T. H. Chan School of Public Health at Harvard University and the University of Southern California. Launched under the auspices of the Ministry of Health and Family Welfare of the Government of India, LASI acts as a thorough source of national data regarding health, economic and social dimensions, as well as the implications of population ageing for individuals aged 45 years and older, including their spouses living in the same household [22]. This initiative encompasses older adults (aged 45 years and above) residing in all the states and union territories throughout India.

The survey methodology employed by LASI utilized a multi-stage stratified area probability cluster sampling approach, consisting of three stages in rural areas and four stages in urban areas. The first stage involved the surveyor selecting a Primary Sampling Unit (PSU) from each state/union territory (UT), followed by the identification of a village (for rural areas) or ward (for urban areas) in the next stage. Ultimately, households were selected by the surveyor in rural contexts, while in urban areas, Census Enumeration Blocks (CEB) were randomly chosen from each locality and households were subsequently identified from the selected CEB [22].

A comprehensive dataset, combining information from both individual and biomarker datasets, was utilized for the analysis. The individual dataset included data on 73,396 individuals, whereas the biomarker dataset contained information on 66,859 individuals. After integrating these datasets and concentrating on older adults specifically, the final analysis retained data on 59,298 individuals aged 45 years and older. Additional details regarding the sampling methodologies and sample sizes can be found in the LASI India Report [9]. In the present analysis, individuals aged 45 years and above were included to align with the LASI study design and to capture both middle-aged (45–59 years) and older adult (60+ years) population. This approach facilitates a life-course perspective by examining early determinants of ageing-related health outcomes, particularly in the Indian context where the burden of non-communicable diseases and functional decline often emerges during midlife [19, 23].

## Data accessibility and ethical considerations

The study is grounded in data from 59,298 older adults aged 45 years and above from LASI Wave-1 (2017–2018). The LASI data is available to the public through a repository at IIPS, necessitating registration and authorization for valid research purposes. Ethical approval for the study was secured from the Indian Council of Medical Research (ICMR) and subsequently ratified by the Institutional Review Board at IIPS. Participants were briefed on the survey’s objectives, as well as the confidentiality and security protocols established for health assessments. Informed consent was acquired at both the household and individual levels, adhering to Human Subject Protection guidelines [22].

## Description of study variables

### Measurement of depressive symptoms

This research primarily concentrated on depressive symptoms, which were evaluated using the 10-item Centre for Epidemiologic Studies Depression Scale (CES-D-10). Participants answered ten questions regarding their experiences over the past week, addressing aspects like concentration difficulties, feelings of depression, low energy levels, fear, loneliness, irritability, exertion, happiness, hope and satisfaction. Each item was rated using a four-point scale such as, (1) rarely or never (less than 1 day), (2) sometimes (1–2 days), (3) often (3–4 days) and (4) most or all of the time (5–7 days) [9]. The CES-D-10 showcased exceptional reliability, with a Cronbach’s alpha of 0.8 [9, 24, 25].

In the present study, depressive symptoms were operationalized using two complementary approaches to align with different analytical frameworks. For the structural equation modeling (SEM) analysis, depression was treated as an underlying continuous latent construct reflected by the CES-D-10 items, enabling the estimation of structural relationships while accounting for measurement error. In contrast, for the ordinal regression analysis, depressive symptoms were categorized into four ordered levels based on response frequency (rarely/never, sometimes, often, most/all of the time), consistent with prior epidemiological applications of CES-D scales. This dual operationalization allows both latent-variable modeling of underlying depressive severity and interpretable estimation of risk gradients across ordered symptom categories [26–29].

## Explanatory variable

The LASI survey gathered information on a range of NCDs, but this study specifically examined the chronic morbidity score. This score was computed based on self-reported NCDs from LASI Wave-1. The four main NCDs considered were diabetes, cancer, chronic respiratory diseases and cardiovascular disease (CVD). Participants self-reported their diagnoses and each disease was coded as a binary variable, ‘1’ for diagnosed and ‘0’ for not diagnosed.

### Other exposure variables

This research investigated a range of socio-demographic, physical, behavioural and health-related factors as exposure variables. The study included the following socio-demographic covariates such as, Age categories: 45–50, 51–55, 56–60, 61–65 and 66+ years, Gender: Male, Female, Education status: Ever attended school, Never attended school, Marital status: Married, Not married, Others (Widowed, Divorced, Separated, Deserted, Live-in relationship), Religion: Hindu, Islam, Others, Place of residence: Rural, Urban, Monthly Per Capita Expenditure (MPCE) Quintile: Poor, Middle, Rich.

Physical limitations were evaluated across three areas, activities of daily living (ADLs), instrumental activities of daily living (IADLs) and mobility challenges. Only issues lasting longer than three months were taken into account. ADLs comprised thirteen everyday self-care tasks such as dressing, walking, bathing, eating, getting in and out of bed, utilizing the toilet, meal preparation, grocery shopping, making phone calls, taking medications, performing housework, managing finances and navigating unfamiliar places. Participants were categorized as, “No ADL difficulty” (no limitations), “1 ADL difficulty” (difficulty in one task), “2+ ADL difficulties” (difficulty in two or more tasks). IADLs included nine instrumental tasks such as using dentures, glasses, hearing aids, walking aids, adapted commodes, shower stools, wheelchairs, neck/back collars, orthotics and prosthetics. Participants were classified as, “IADL absent” (no difficulties), “IADL present” (any difficulty present in IADLs). Mobility challenges encompassed nine movement-related challenges, including walking long distances, sitting for prolonged periods, rising from a chair, climbing stairs, bending, reaching, pulling/pushing, lifting heavy items and picking up objects. Participants were categorized as, “No mobility difficulty”, “1 mobility difficulty”, “2+ mobility difficulties”.

Self-rated health (SRH) was evaluated through the inquiry like, “Overall, how would you describe your health in general?” which provided responses, 1 = Good, 2 = Poor. Smoking status was established based on self-disclosed tobacco usage, “Yes” (0): Currently smokes, “No” (1): Never smoked or previously a smoker. Alcohol consumption was gauged by assessing alcohol intake over the previous three months, “Yes” (0): Consumed alcohol in the past three months, “No” (1): Did not consume alcohol in the past three months. Sleep quality was measured through three inquiries regarding the difficulty of falling asleep, experiencing night-time awakenings and waking up too early in the morning. Inadequate sleep was categorized as “Yes” if participants reported sleep difficulties 3–4 nights/week or more, and “No” if they indicated never or rarely (1–2 nights/week). Physical activity was assessed by inquiring about engagement in moderate activities such as cleaning, doing laundry, fetching water or wood, gardening, biking, walking, dancing, or performing stretching exercises. Participants were classified as, “Physically active” (0): Participated in these activities daily or more than once a week and “Physically inactive” (1): Engaged in these activities never or only once a month. Body Mass Index (BMI) was derived from self-reported weight and height (kg/m²). BMI was categorized according to Asia-Pacific (India) criteria such as, Underweight (< 18.5), Normal weight (18.5–22.9), Overweight (23.0-24.9) and Obese (≥ 25). This extensive array of exposure variables facilitated a comprehensive study of factors that affect the study outcomes.

### Statistical analysis

Data were analyzed using the LASI Wave 1 dataset (2017-18), which included a nationally representative sample of 59,298 older adults aged 45 years and above. Analyses were performed using Stata 17.0 and Jupyter notebook (including Python). Descriptive statistics were computed to summarize socio-demographic characteristics, health status and behavioural factors.

## Latent variable modeling: EFA and CFA

An exploratory factor analysis (EFA) was first conducted on selected health and behavioural indicators (ADL, IADL, mobility, smoking, alcohol use, diabetes, CVD, etc.) to reduce dimensionality and identify underlying latent constructs. The Kaiser-Meyer-Olkin (KMO) measure and Bartlett’s test of sphericity were used to assess sampling adequacy and suitability for factor analysis [30]. The EFA was based on the principal-axis factoring extraction method with varimax rotation, retaining factors with eigenvalues > 1 according to the Kaiser criterion and visual inspection of the scree plot.

Mathematically, the factor model can be expressed as:

$$\:x=\:{\Lambda\:}\mathrm{f}\:+\:\in\:\:$$

where, * x* is the vector of observed standardized variables (i.e., ADL, IADL, NCDs), ‘f’ represents latent factors, $\:{\Lambda\:}\:$denotes the matrix of factor loadings and $\:\in\:$ is the vector of unique variances (measurement errors).

The extracted factors were conceptually labelled as functional limitations (Factor 1), lifestyle behaviours (Factor 2) and NCDs (Factor 3), consistent with prior literature linking these domains to depression [31]. Following EFA, a Confirmatory Factor Analysis (CFA) was conducted to validate the measurement model. The CFA tested whether the observed variables adequately loaded onto their respective latent constructs using the maximum likelihood estimation method. Model fit was evaluated using multiple performance indices, including the Comparative Fit Index (CFI), Tucker-Lewis Index (TLI), Root Mean Square Error of Approximation (RMSEA) and Standardized Root Mean Square Residual (SRMR). Acceptable model fit was defined as CFI ≥ 0.90, TLI ≥ 0.85, RMSEA ≤ 0.08 and SRMR ≤ 0.05 [32, 33].

### Structural Equation Modeling (SEM)

Structural equation modeling is a prominent multivariate method for exploring both the direct and indirect impacts of the relationships between observed and latent variables. In SEM, an observed variable is one that has been directly quantified and plays a role in forming a latent variable. Conversely, latent variables are those that are not directly measurable. The SEM framework clarifies intricate relationships among variables by employing a path model or analysis to elucidate effects derived from both observed and latent variables [34, 35]. This technique distinctly identifies the connections between cause-and-effect variables. The objective of SEM is to extend a CFA model to evaluate the interrelationships among latent variables and their diverse influences on one another. SEM encapsulates a linear structural relationship into a measurement model (CFA model) and a structural model [36].

The validated measurement model was extended to SEM to examine the direct and indirect pathways linking functional limitations, lifestyle behaviours and NCDs burden to depressive symptoms. The SEM integrates both measurement and structural components, first is, measurement model that defines how latent constructs are represented by observed indicators and second is, structural model that specifies the directional relationships among latent constructs.

Formally, the SEM can be represented as:

$$\:{\upeta\:}=\:\mathrm{B}{\upeta\:}+\:{\Gamma\:}{\upxi\:}+\:{\upzeta\:}$$

and, $$\:\mathrm{y}={{\Lambda\:}}_{y}{\upeta\:}+\:{\in\:}_{y}\:$$  

and, $$\:x=\:{{\Lambda\:}}_{x}{\upxi\:}+\:{\delta\:}_{x}$$

where, η denotes endogenous latent variables (such as, depression), ξ denotes exogenous latent variables (such as, functional limitations, lifestyle, NCDs), B and Γ represents matrices of structural coefficients, ζ shows structural disturbances (errors) and $\:{{\Lambda\:}}_{y}$ and $\:{{\Lambda\:}}_{x}$ denotes factor loading matrices for observed variables. Path coefficients (β) represent standardized effects, allowing comparison of the relative strength of associations. Statistical significance was assessed at *p* < 0.05. Model adequacy was verified using the same goodness-of-fit indices as in CFA. SEM is particularly suited to the present study because it allows simultaneous estimation of multiple interdependent relationships and decomposes total effects into direct and indirect components [27, 37].

In this framework, depression was specified as a continuous latent endogenous construct, reflecting the underlying severity of depressive symptoms derived from CES-D responses, which is consistent with latent variable modeling approaches commonly used in psychiatric epidemiology [27].

### Ordinal regression modeling

A proportional odds ordinal logistic regression was estimated to validate the SEM findings and to quantify the influence of key predictors on depressive symptom severity. The dependent variable represented ordered categories of depressive symptoms i.e., rarely/never, sometimes, often and most/all of the time.

The cumulative logit model is defined as:

$$\:\mathrm{ln}(\frac{P(Y\le\:j)}{P(Y>j)})=\:{\alpha\:}_{j}-\:{\beta\:}_{1}{X}_{1}-\:{\beta\:}_{2}{X}_{2}-.\dots\:\dots\:.\:.-{\beta\:}_{k}{X}_{k}$$

for thresholds j = 1,2,3….k where, * Y* is the ordinal depression outcome, $\:{\alpha\:}_{j}$ are category-specific intercepts and $\:{\beta\:}_{k}$ denote regression coefficients for predictors $\:{X}_{k}$. Positive β values indicate higher odds of reporting more severe depressive symptoms. Model diagnostics included Brant tests for the proportional odds assumption and likelihood-ratio tests for overall model significance [28]. Moreover, for assessing model robustness, a bootstrap resampling procedure (1,000 iterations) was applied to estimate bias-corrected 95% confidence intervals for SEM path coefficients. Sensitivity analyses were performed by excluding cases with missing data and re-estimating the model to ensure stability of parameter estimates, where all statistical tests exist with a significance level of *p* < 0.05.

Also, unlike the SEM framework, the ordinal regression model treated depressive symptoms as an ordered categorical outcome. This approach was used as a complementary validation strategy to assess whether the direction and magnitude of associations observed in SEM were consistent when depressive symptoms were expressed in discrete severity categories.

## Results

Table 1 represents the socio-demographic and health-related characteristics of 59,298 older adults aged 45 years and above included in the study. The prevalence of depressive symptoms varied considerably across four categories. There were 26.9% of respondents who experienced depressive symptoms for sometimes (1–2 days), while about 9.4% reported feeling depressed for often (3–4 days) and 3% experienced depressive feelings for most or all of the time (5–7 days). These distributions suggest that nearly 40% of older adults reported some level of depressive symptomatology, indicating a notable burden of psychological distress among ageing population in India. Among four major NCDs, diabetes was reported by 12.7% of respondents, CVD by 3.6% and chronic respiratory diseases by 5.6%. In context of gender, there were higher proportion of females (53.7%) than males (46.3%). Approximately 26.9% of respondents were aged 66 years and above, followed by 25.2% in the 45–50 age group. More than half of the respondents resided in rural areas (65.6%), where three-fourths (74.9%) were married. There were 47% of respondents having never attended school and majority were Hindus (73.2%), followed by Islam (11.7%) and other religions (15.1%) and nearly 40% of respondents were in the poor MPCE quintile. About 24.9% had two or more ADL limitations and 50.6% had two or more mobility limitations, reflecting a substantial burden of functional impairment. Similarly, 38.7% had IADL limitations, indicating challenges in performing more complex daily tasks. Although, 36.7% of respondents were smoked and 17.9% were consumed alcohol. Only 31.2% were physically active, while a striking 68.8% were inactive. Additionally, 90.8% of older adults reported being socially inactive, reflecting limited engagement in social or community activities, an important psychosocial risk factor for depression. Overall, the descriptive statistics indicate a high burden of health and functional challenges among older adults in India.

**Table 1:** Socio-demographic characteristics and health profile of older adults aged 45 years and above in India, LASI Wave 1 (2017-18)

Variables	Category	Total (*n* = 59,298)	
		*n*	%
Outcome Variable			
Depressive symptoms	Rarely or never (less than 1 day)	36,072	60.8
	Sometimes (1 or 2 days)	15,922	26.9
	Often (3 or 4 days)	5,554	9.4
	Most or all of the time (5–7 days)	1,750	3.0
Exposure variable			
Diabetes	No	51,743	87.3
	Yes	7,555	12.7
Cancer	No	58,917	99.4
	Yes	381	0.6
Chronic respiratory diseases	No	55,973	94.4
	Yes	3,325	5.6
CVD	No	57,160	96.4
	Yes	2,138	3.6
Co-variates			
Age of respondents	45–50	14,915	25.2
	51–55	9,754	16.4
	56–60	9,389	15.8
	61–65	9,268	15.6
	66 and above	15,972	26.9
Gender	Male	27,448	46.3
	Female	31,850	53.7
Place of residence	Rural	38,923	65.6
	Urban	20,375	34.4
Marital status	Married	44,435	74.9
	Unmarried	699	1.2
	Others	14,164	23.9
Education	Never attended school	27,882	47.0
	Attended school	31,416	53.0
Religion	Hindu	43,386	73.2
	Islam	6,946	11.7
	Others	8,966	15.1
MPCE quintile	Poor	23,722	40.0
	Middle	11,989	20.2
	Rich	23,587	39.8
ADL limitations	0	38,258	64.5
	1	6,251	10.5
	2+	14,789	24.9
Mobility limitations	0	23,209	39.1
	1	6,106	10.3
	2+	29,983	50.6
IADL status	Absent	36,360	61.3
	Present	22,938	38.7
Poor sleep	No	49,022	82.7
	Yes	10,276	17.3
Self-rated health	Good	36,998	62.4
	Poor	22,300	37.6
Smoking	No	37,562	63.3
	Yes	21,736	36.7
Consumed alcohol	No	48,663	82.1
	Yes	10,635	17.9
Physical activity	Active	18,504	31.2
	Inactive	40,794	68.8
Social activity status	Active	5,440	9.2
	Inactive	53,858	90.8
BMI	Underweight	10,875	18.3
	Normal	21,990	37.1
	Overweight	9,036	15.2
	Obese	17,397	29.3

***MPCE: Monthly Per Capita Expenditure; SRH: Self-rated health; ADL: Activities of Daily Living; IADL: Instrumental activities of daily living.

Table 2 provides bridge to identify latent constructs underlying the observed health and behavioural indicators, an Exploratory Factor Analysis was conducted using data from LASI Wave 1. The Kaiser Meyer Olkin (KMO) measure of sampling adequacy was 0.603, indicating an acceptable level of intercorrelation among variables for factor analysis. Bartlett’s test of sphericity was highly significant (χ²= 34,646.32, *p* < 0.001), confirming that the correlation matrix was not an identity matrix and was therefore suitable for factor extraction. Based on eigenvalues greater than one and examine of the scree plot, a three-factor solution was retained, explaining the major dimensions of variability among the selected indicators. The exploratory factor analysis identified three distinct latent constructs corresponding to functional limitations (Factor 1), lifestyle behaviours (Factor 2) and NCDs (Factor 3). Variables with strong loadings clustered meaningfully within these domains, supporting the conceptual framework of the study. Only key loadings are highlighted here, while detailed factor loadings are presented in Table 2.

Factor 1 (functional limitations) was defined by strong positive loadings on ADL status (0.686) and mobility status (0.668) and Factor 2 (lifestyle behaviours) showed high positive loadings for smoking (0.630) and alcohol consumption (0.607), indicating that these behaviours co-occur and define a latent lifestyle domain related to substance use. Factor 3 (NCDs) was characterized by positive loadings on diabetes (0.380) and CVD (0.244), representing the chronic health conditions typical of the NCDs burden among older adults.

Variables with factor loadings below the commonly accepted threshold of 0.40 were considered weak indicators and were not retained within the latent constructs. This threshold is widely recommended in multivariate analysis to ensure adequate contribution of observed variables to underlying latent factors [38, 39]. Accordingly, variables such as cancer, chronic respiratory diseases, physical activity, poor sleep and social activity status did not demonstrate sufficient loading strength on any of the extracted factors and were therefore excluded from the final factor structure.

Although these variables are theoretically relevant to depression and ageing, their weak empirical loadings indicate that they did not cluster meaningfully with the identified latent domains in this dataset. Retaining such variables could have introduced noise and reduced the interpretability and statistical robustness of the latent constructs. Therefore, their exclusion reflects an empirically driven decision to preserve construct coherence and improve model stability [40]. But, to maintain conceptual completeness, selected variables such as sleep quality, physical activity and social activity status were retained as independent covariates in subsequent regression analyses. This approach ensures that their potential influence on depressive symptoms is still accounted for, even though they were not included within the latent factor structure.

**Table 2:** Exploratory Factor Analysis (EFA) showing Factor Loadings and Sampling Adequacy for indicators related to Depression among older adults, LASI Wave 1 (2017-18)

Variable	Factor1	Factor2	Factor3
Diabetes	0.065	-0.070	0.380
Cancer	-0.018	-0.002	0.056
Chronic respiratory diseases	-0.117	0.060	0.086
CVD	-0.024	0.022	0.244
Smoking	-0.125	0.630	-0.113
Consumed alcohol	-0.001	0.607	-0.019
Physical activity	0.125	0.172	-0.169
Poor sleep	-0.241	0.024	0.082
Social activity status	0.113	0.054	0.085
IADL limitations	-0.044	0.046	-0.422
ADL status	0.686	-0.036	0.094
Mobility status	0.668	-0.046	-0.087

**KMO: 0.6029754555740798 and Bartlett’s test: χ² = 34646.31731539078, *p* < 0.0001

Table 3 presents the model incorporated three latent constructs derived from the preceding Exploratory and Confirmatory Factor Analyses, representing distinct but interrelated dimensions of health and behaviour. The structural equation modeling demonstrated significant relationships among the latent constructs. Functional limitations emerged as the strongest predictor of depressive symptoms, while lifestyle behaviours and NCDs burden also showed significant associations. Although the association between NCDs burden and functional limitations was statistically significant, its direction was contrary to theoretical expectations and is interpreted with caution. The functional limitations factor was negatively associated with the NCDs factor (β= -0.280, *p* < 0.001). While this association is statistically significant, its direction is contrary to established theoretical expectations and should be interpreted with caution. This unexpected finding may reflect complexities related to measurement model specification or underlying heterogeneity in the study population rather than a true inverse relationship.

Overall, the model supports the role of functional health as a key pathway linking physical and behavioural factors to depression. These findings highlight the intertwined effects of physical function, NCDs status and health behaviours in shaping the mental health of India’s ageing population. Further, the overall goodness of fit statistics for the Structural Equation Modeling was shown in supplementary table (Table S1), demonstrated an acceptable to good fit to the observed data across multiple indices.

**Table 3:** Structural Equation Modeling (SEM) showing Standardized Path Coefficients and Significance Levels for Depression among older adults in India (LASI Wave 1, 2017-18)

Outcome	Predictor	Estimate (β)	Std. Error	z-value	*p*-value
Functional Limitations factor	NCDs factor	-0.280	0.078	-3.562	0.000367
Functional Limitations factor	Lifestyle factor	0.004	0.007	0.562	0.57384
Diabetes	NCDs factor	1	-	-	-
Smoking	Lifestyle factor	1	-	-	-
Consumed alcohol	Lifestyle factor	1.362	0.116	11.696	0
IADL limitations	Functional Limitations factor	1	-	-	-
ADL status	Functional Limitations factor	6.890	0.245	28.099	0
Mobility status	Functional Limitations factor	10.483	0.417	25.102	0
Depression	NCDs factor	0.301	0.051	5.848	0
Depression	Lifestyle factor	0.092	0.018	5.046	0
Depression	Functional Limitations factor	2.052	0.083	24.535	0
Functional Limitations factor	Functional Limitations factor	0.004	0.0003	12.836	0
Lifestyle factor	Lifestyle factor	0.050	0.004	11.507	0
Lifestyle factor	NCDs factor	-0.004	0.0005	-9.058	0
NCDs factor	NCDs factor	0.009	0.002	3.761	0.000169
ADL status	ADL status	0.502	0.006	73.819	0
Consumed_alcohol	Consumed_alcohol	0.054	0.007	6.787	0
Depression	Depression	0.593	0.003	167.854	0
Diabetes	Diabetes	0.102	0.002	40.888	0
IADL limitations	IADL limitations	0.232	0.001	170.252	0
Mobility status	Mobility status	0.340	0.014	23.744	0
Smoking	Smoking	0.181	0.004	41.213	0

**Fig. 2:**
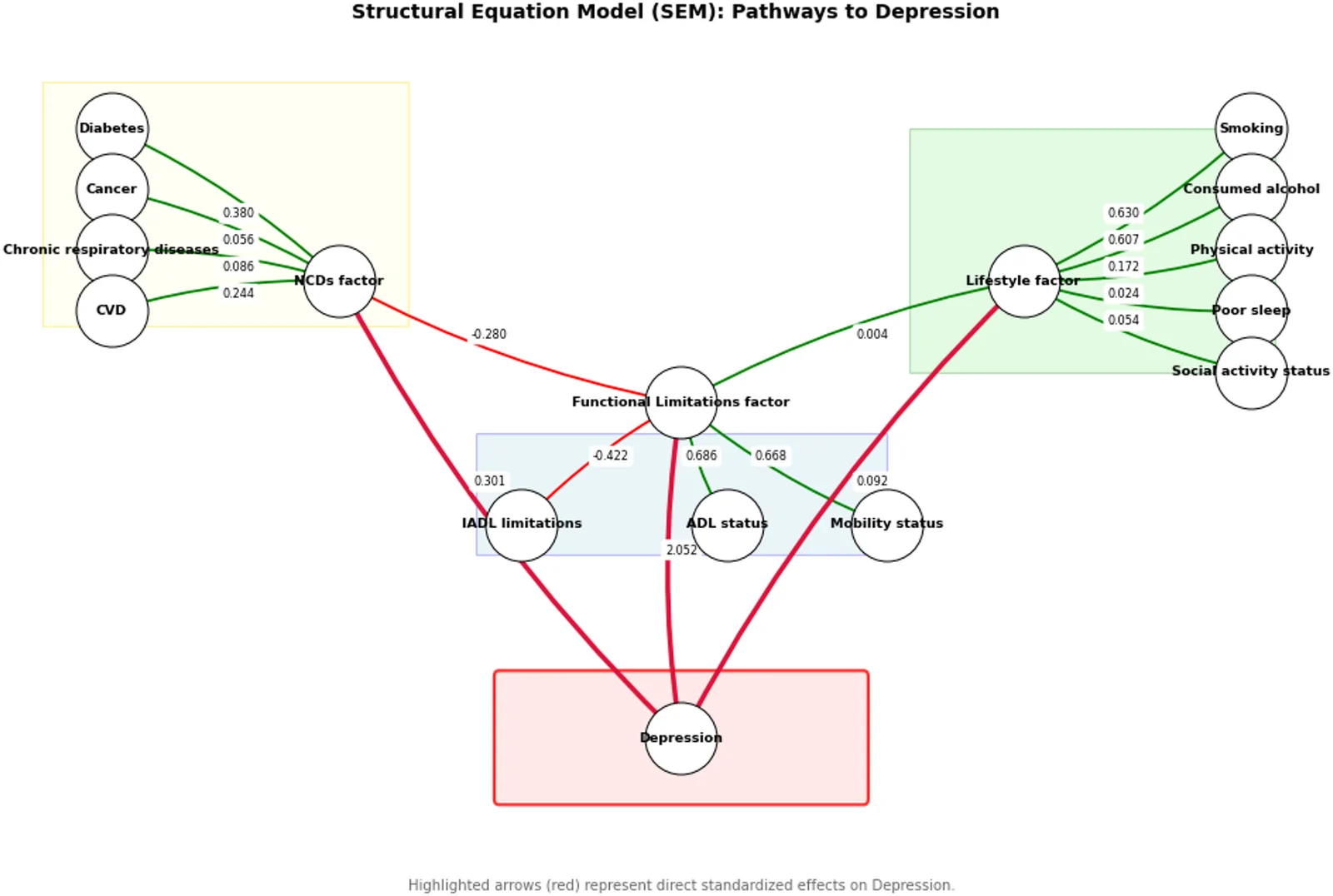
Structural Equation Modeling (SEM) depicting direct and indirect pathways from Functional Limitations, Lifestyle Behaviours and NCDs to Depression among Older Adults in India

Figure 2 illustrates the analytical framework through SEM depicting the direct and indirect pathways from latent health domains i.e., functional limitations, lifestyle behaviours and NCDs to depression among older adults in India. The model was developed based on the three-factor structure extracted from EFA and subsequently validated through SEM estimation.

The diagram visually demonstrates that all three latent constructs had statistically significant direct effects on depression, as indicated by the highlighted red arrows. The functional limitations factor exhibited the strongest positive association with depression (β = 2.052, *p* < 0.001), suggesting that declines in physical functioning, measured through ADL, mobility and IADL limitations are key determinants of depressive symptoms in later life. The NCDs factor also showed a positive direct association with depression (β = 0.301, *p* < 0.001), indicating that chronic conditions such as diabetes and cardiovascular disease contribute to increased depressive symptomatology. Additionally, the lifestyle behaviours factor had a smaller but statistically significant direct effect (β = 0.092, *p* < 0.001), implying that lifestyle patterns related to smoking, alcohol consumption and physical inactivity influence depressive outcomes.

Measurement pathways (green arrows) show strong positive loadings of observed indicators onto their respective latent constructs, confirming good internal consistency within each domain particularly for functional indicators (ADL = 0.686 and mobility = 0.668) and lifestyle indicators (smoking = 0.630 and alcohol consumption = 0.607). Overall, the SEM diagram highlights a multidimensional causal structure in which depression among older adults emerges primarily from functional impairment, with additional contributions from NCDs burden and lifestyle risk behaviours. This visualization underscores the interdependence of physical, behavioural and health domains in shaping mental health among older adults.

**Fig. 3:**
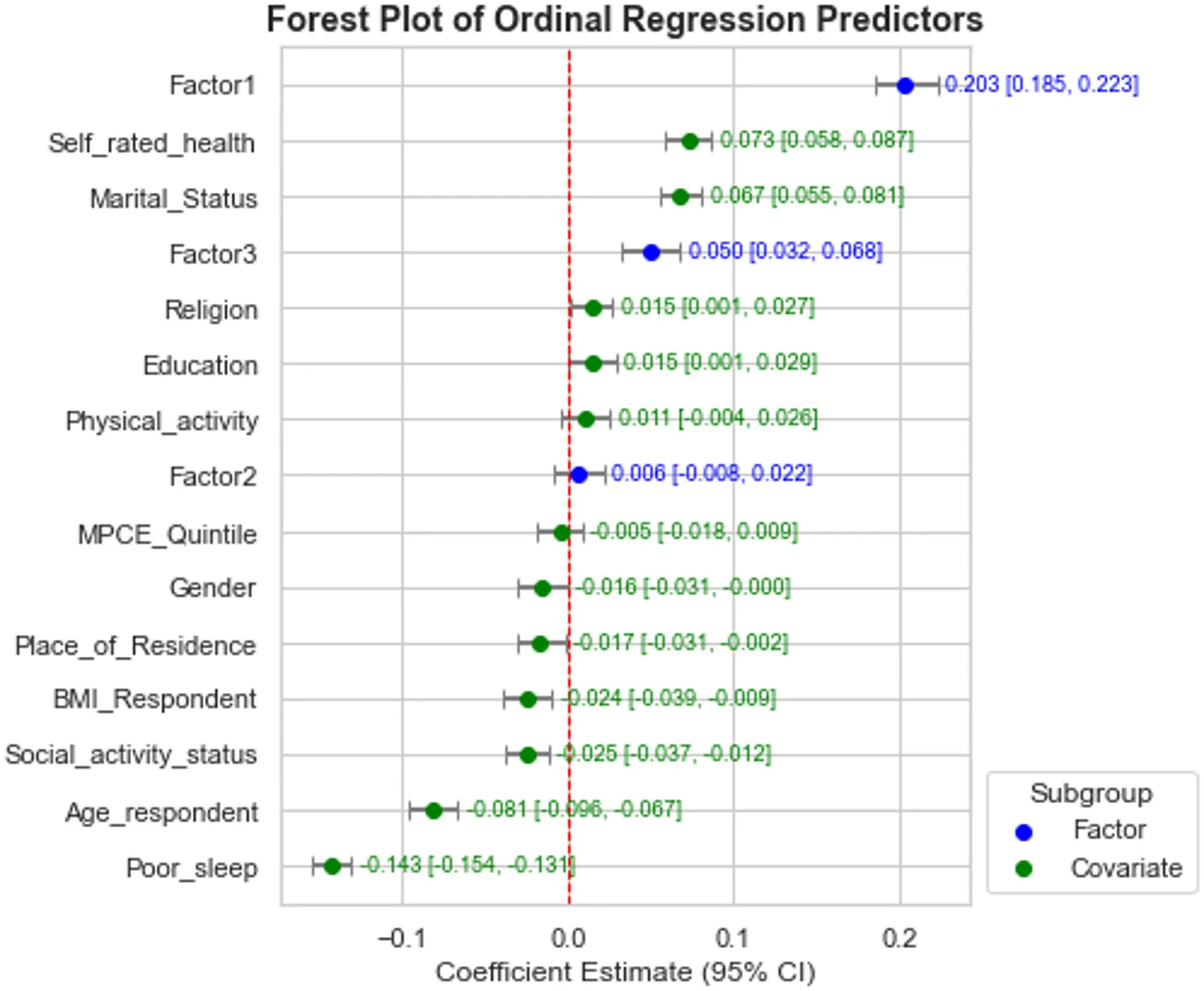
Forest plot of ordinal regression predictors of depression among older adults in India

Figure 3 shows the forest plot of ordinal regression predictors with coefficient estimates and their 95% confidence intervals. Positive coefficients indicate a direct association with the dependent variable, while negative coefficients indicate an inverse association. Predictors were grouped into two categories i.e., latent factors derived from confirmatory factor analysis (blue points) and individual covariates (green points). The vertical dashed red line at zero represents the null effect line, where coefficients crossing this line indicate non-significant associations. Consistent with the analytical framework, depression was modeled as a latent continuous construct in SEM, whereas ordinal regression modeling analyses used categorized depressive symptom levels to facilitate interpretation of severity gradients.

Among all predictors, factor 1 shows the highest positive coefficient (β = 0.203, 95% CI: 0.185–0.223), indicating the strongest positive relationship with the depression. Other strong predictors with positive associations include self-rated health (β = 0.073, 95% CI: 0.058–0.087), marital status (β = 0.067, 95% CI: 0.055–0.081), and factor 3 (β = 0.050, 95% CI: 0.032–0.068).

Thus, the ordinal regression analysis further confirmed the significant associations between functional limitations, lifestyle factors and depressive symptoms. Functional impairment variables showed strong positive associations with higher levels of depression. Most behavioural and health-related variables were associated with increased odds of depressive symptoms, although some coefficients (e.g., poor sleep) exhibited unexpected directions and are discussed further in the Discussion section. In short, poor sleep exhibited a statistically significant negative coefficient (β= -0.143), indicating an inverse association with depressive symptoms in the adjusted model. However, this direction is contrary to theoretical expectations and should be interpreted with caution, as it may reflect underlying statistical or model-related complexities rather than a true protective effect.

**Fig. 4:**
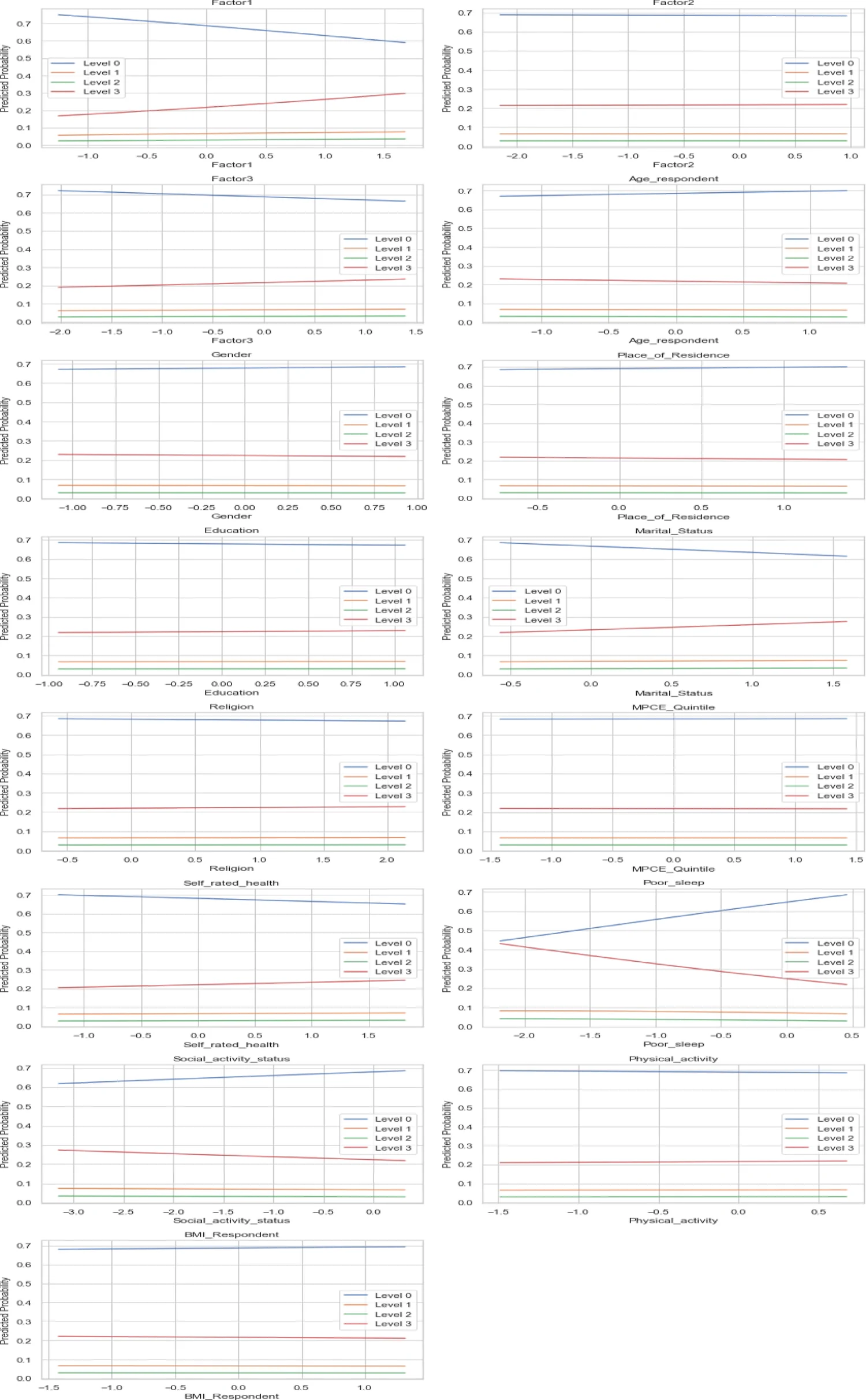
Predicted Probabilities of depressive symptoms severity across selected predictors among older adults in India

Figure 4 displays the predicted probabilities of experiencing different levels of depressive symptoms (rarely/never, sometimes, often and severe/most or all of the time) across a range of predictors derived from the ordinal regression modeling. The coloured lines represent the four depression categories i.e., (blue= level 0) for ‘rarely or never’ depressive symptoms, (orange= level 1) for ‘sometimes’ depressive symptoms, (green= level 2) for ‘often’ depressive symptoms and (red= level 3) for ‘severe’ depressive symptoms.

This figure highlights that functional limitations (factor 1), NCDs burden (factor 3), marital status, poor self-rated health and education predictor exhibit gradual upward shifts in the predicted probability of higher depressive symptoms, followed by the religion and lack of physical activity. Together, these results suggest that the risk of depression among older adults is more strongly influenced by physical functioning and NCDs-related factors than by lifestyle or demographic characteristics.

## Discussion

The results of this study showed strong significant factors frequently neglected and overlooked in the public health context within India. With the large sample of 59,298 older adults in India aged 45 years and above, observation of this study says that nearly 40% of respondents reported some level of depressive symptoms, with 9.4% experiencing symptoms “often” and 3% experiencing symptoms “most or all of the time”, reflecting a substantial burden of subclinical and clinical distress. This estimate exceeds several earlier Indian meta-analyses (i.e., ~ 34.4%) and likely reflects the broader symptom-based measurement approach rather than strict diagnostic criteria, thereby capturing the hidden cause of depression in ageing population [41, 42].

In contrast to significant previous research, which predominantly focused on the prevalence of multimorbidity dyads and triads while investigating solely the linearly correlated risk factors [43, 44], and still NCDs are increasing silently, along with depression which is continuously spreading its wings in the society, that shows there is something hidden which is not clearly explored and highlighted. The findings from this study employed innovative EFA and SEM as a rigorous methodology to unveil the underlying determinants of depression among older adults with NCDs across diverse socio-economic and demographic risk factors. A major contribution of this study lies in identifying three latent domains such as, functional limitations, NCDs burden and lifestyle behaviours through EFA and validating their structural relationships using SEM. Among these, functional limitations emerged as the strongest predictor of depressive symptoms, consistent with extensive gerontological literature demonstrating that impairments in activities of daily living (ADL/IADL) and mobility significantly elevate depression risk [45–48]. The magnitude of this association highlights that loss of independence, reduced functional capacity [49] and feelings of helplessness [50] are central pathways linking physical health to mental well-being in later life. Thus, the association is likely bidirectional and self-reinforcing among older adults.

The use of both SEM and ordinal regression modeling frameworks reflects a complementary analytical strategy rather than inconsistency. While SEM captures the latent continuous nature of depressive symptomatology and allows for the estimation of complex structural pathways, the ordinal regression modeling provides an interpretable validation of these associations across ordered severity categories. This combined approach has been recommended in epidemiological and psychiatric research to enhance robustness and interpretability of findings when working with psychometric scales such as CES-D [26, 28, 29].

And important thing is that the findings support prior evidence that functional impairment often mediates the relationship between chronic disease and depression [10, 51, 52]. While NCDs burden showed a statistically significant direct association with depression, its comparatively smaller effect size suggests that its influence may operate partially through functional decline [49]. This aligns with studies in India showing that comorbidity or multimorbidity increases depression risk, particularly when accompanied by disability and reduced quality of life [53].

An important and somewhat unexpected finding in this study is the negative association between the NCDs factor and functional limitations (β= -0.280). This result appears counterintuitive, as a substantial body of literature indicates that chronic conditions generally increase the risk of functional decline and disability in older adults. Therefore, this association should not be interpreted as evidence of a protective effect of NCDs on functional health.

Several methodological and contextual explanations may account for this pattern. First, the NCDs construct in the present study was derived from a limited number of self-reported conditions, with relatively modest factor loadings. This may not fully capture the cumulative burden, severity or duration of chronic illness, thereby affecting the direction and magnitude of associations. Second, it is possible that individuals reporting NCDs in this dataset are more likely to be diagnosed and treated, reflecting better access to healthcare, which may partially mitigate functional decline compared to undiagnosed or untreated individuals [17]. Third, the observed relationship may reflect a suppression arising from inter-correlations among latent constructs within the SEM framework. Importantly, the cross-sectional design of the study limits causal inference and reverse or bidirectional relationships between NCDs and functional limitations cannot be ruled out. Prior research consistently demonstrates that multimorbidity contributes to disability and functional impairment in ageing population [19, 54]. Therefore, the present finding should be interpreted cautiously and viewed as indicative of underlying model complexity rather than a deviation from established epidemiological evidence.

However, the relatively modest loading of NCDs indicators (< 0.40) and weaker effect size may reflect heterogeneity among disease types, measurement error (self-report) or insufficient number of disease indicators in the factor. Cancer and chronic respiratory disease did not load well and were excluded as weak indicators in the EFA, which may have reduced the comprehensiveness of the NCD latent domain. Empirical studies have similarly documented associations between NCDs and depression in older population [51]. An important methodological consideration in this study relates to the exclusion of several theoretically relevant indicators from the latent constructs due to weak factor loadings. Variables such as cancer, chronic respiratory diseases, physical activity, sleep quality and social activity did not meet the minimum loading threshold and were therefore not retained in the EFA-derived factor structure [40]. While this decision improves the internal consistency and statistical validity of the constructs, it may have implications for content validity, as these variables are known to be associated with depression among older adults.

This highlights a common trade-off in latent variable modeling between empirical fit and theoretical comprehensiveness [27, 39]. In the present study, priority was given to achieving a parsimonious and statistically stable factor structure. Nevertheless, the inclusion of these variables in the ordinal regression modeling analysis partially mitigates this limitation by ensuring their independent associations with depressive symptoms are examined. Future research may explore alternative measurement models or incorporate additional indicators to better capture these dimensions within latent constructs.

Lifestyle behaviours exhibited a weaker but statistically significant association with depressive symptoms, consistent with evidence linking smoking, alcohol use and sedentary lifestyles to mental health outcomes through both biological and psychosocial pathways [55]. Prior research shows that unhealthy behaviours are risk factors for depression, both by promoting biochemical dysregulation and psychosocial pathways (i.e., social stigma, poor health perception) [23, 56]. The limited role of physical activity within the latent construct suggests potential measurement heterogeneity or context-specific behavioural patterns, which warrants further investigation.

Another important and unexpected finding in the present study is the inverse association between poor sleep and depressive symptoms observed in the ordinal regression modeling analysis. This result is contrary to a well-established body of evidence demonstrating that sleep disturbances are a significant risk factor for depression across age groups, including older adults [57, 58]. Therefore, this finding should not be interpreted as indicating a protective effect of poor sleep.

Several methodological explanations may account for this counterintuitive result. First, a suppressor effect may be present, whereby the inclusion of strongly correlated predictors such as functional limitations, self-rated health and NCDs burden alters the direction of the sleep coefficient when all variables are simultaneously included in the model. In such cases, a variable that is positively associated with the outcome in bivariate analysis may appear negatively associated in multivariable models due to shared variance with other predictors. Secondly, multicollinearity among health-related variables may contribute to instability in coefficient estimates. Sleep disturbance is conceptually and empirically linked with physical health, functional impairment and psychological distress [58], and its independent effect may be difficult to disentangle when these overlapping constructs are included in the same model. Third, potential model specification issues may underlie this finding. In the present study, poor sleep category was included as an observed independent variable rather than being incorporated into a broader latent construct of health or well-being. Given its multidimensional nature, sleep quality may interact with both physical and psychological domains in ways not fully captured by the current model structure. Hence, prior research consistently demonstrates a positive association between sleep disturbances and depression. Studies have shown that individuals with insomnia or poor sleep quality have significantly higher risk of developing depressive symptoms [59, 60]. It is also estimated that a large proportion of individuals with depression report co-occurring sleep disturbances, highlighting the bidirectional relationship between these conditions [61]. Therefore, the inverse association observed in this study likely reflect suppressor effect or model-related complexities rather than a true epidemiological relationship. Future research using longitudinal data and alternative modeling approaches may help clarify the role of sleep disturbances in depression among older adults.

The role of sociodemographic factors observed in this study is broadly consistent with existing literature [13]. Poor self-rated health, marital disruption and social inactivity were associated with higher depressive symptoms, highlighting the importance of subjective health perception and social engagement in shaping mental well-being [10, 62]. The negative association with age may reflect survival bias or underreporting among older cohorts, a phenomenon previously documented in ageing research [63].

From a methodological perspective, this study advances existing research by integrating exploratory factor analysis, structural equation modeling and ordinal regression modeling within a unified analytical framework. While SEM enables the modeling of latent constructs and complex pathways, ordinal regression modeling complements this by providing interpretable estimates across ordered categories of depressive severity. This combined approach enhances both robustness and interpretability, particularly when working with psychometric scales such as CES-D [33, 64].

From a policy and public health perspective, the findings enlighten the need to prioritize functional health in ageing population. Interventions aimed at maintaining mobility, independence and daily functioning such as rehabilitation services, assistive technologies and community-based care may yield substantial mental health benefits [65]. Additionally, the significant role of NCDs burden highlights the importance of integrated care models that simultaneously address physical and mental health conditions. Routine screening for depression among individuals with chronic diseases and vice versa, should be incorporated into primary healthcare systems in India. Strengthening primary healthcare systems to incorporate routine mental health assessment among individuals with functional limitations and chronic diseases could facilitate early identification and intervention. Furthermore, the high prevalence of depressive symptoms suggests that a large proportion of older adults remain undiagnosed or untreated, consistent with the “iceberg phenomenon” in epidemiology [66]. This calls for strengthened mental health surveillance, improved access to geriatric mental health services and community-level interventions to reduce stigma and enhance early detection.

Hence, this study demonstrates that functional limitations are the most critical determinant of depressive symptoms among older adults in India, with additional contributions from chronic disease burden and lifestyle behaviours. By combining latent variable modeling with regression-based validation, the study provides a comprehensive and methodologically rigorous framework for understanding depression in later life. Future research should build on these findings using longitudinal data and advanced modeling techniques, including mixture models and machine learning approaches, to further uncover hidden patterns and trajectories of mental health risk in ageing population.

### Future implications

The findings underscore the importance of incorporating functional health assessment into routine geriatric care to enable early identification of individuals at risk of depression. Regular screening of ADL, mobility and IADL within primary and community healthcare settings can support timely and targeted interventions. Integrating chronic disease management with physical rehabilitation and mental health services may further enhance outcomes for older adults. In addition, public health strategies should prioritize modifiable lifestyle factors, including reducing tobacco and alcohol use, promoting physical activity and strengthening social engagement through community-based programs. The study highlights the interconnected roles of functional health, NCDs burden and behavioural factors in shaping late-life depression. The complexity observed in these relationships suggests the need for refined models that allow for overlapping and potentially bidirectional pathways. Future research should employ longitudinal designs to establish causal relationships and incorporate broader health dimensions, such as cognitive function, nutrition and biological markers, to better understand the mechanisms underlying depression in ageing population.

### Limitations of the study

This study possesses several notable strengths that enhance the validity and generalizability of its findings. However, this study has several limitations that should be considered when interpreting the findings. First, the cross-sectional design precludes causal inference and the observed associations and structural pathways should not be interpreted as evidence of directionality. The relationship identified may be bidirectional or influenced by unmeasured temporal dynamics. Secondly, the analysis relies on self-reported measures, including NCDs status, functional limitations (ADL, IADL, mobility), sleep quality and social participation. These variables may be subject to recall bias, reporting bias or misclassification, which could influence the estimated associations. Third, certain modelling challenges warrant consideration. The unexpected inverse association observed for sleep and the negative pathway between NCDs and functional limitations may reflect suppressor effects, multicollinearity or model specification issues. Lastly, residual confounding cannot be ruled out. Despite these limitations, the study offers valuable insights into the complex interrelationships between functional health, chronic disease burden, lifestyle behaviour and depressive symptoms. Future research using longitudinal data and alternative advanced modelling approaches is needed to validate these findings and better establish causal pathways.

## Conclusion

In a nutshell, this study highlights the significant role of functional limitations, non-communicable disease burden and lifestyle behaviours in shaping depressive symptoms among older adults in India. Among these factors, functional limitations demonstrated the strongest association with depressive symptom severity, suggesting that physical functioning is a key component of mental well-being in later life. While NCDs burden and lifestyle behaviours were also associated with depression, their effects appear to operate both directly and in conjunction with functional health. These findings highlight the importance of adopting integrated and multidimensional approaches to ageing and mental health.

From a public health perspective, the results suggest that interventions aimed at preserving functional ability, alongside effective management of chronic conditions and promotion of healthy behaviour, may contribute to improved mental health outcomes. Overall, this study provides a comprehensive analytical framework for understanding the complex interplay between physical health and depression in ageing population and highlight the need for longitudinal and intervention-based research to further clarify these pathways.

## Supplementary Information

Below is the link to the electronic supplementary material.

Supplementary Material 1

## Data Availability

The present study uses data from a secondary source that is openly accessible in the public domain at (https:/g2aging.org).

## Abbreviations

ADL: Activities of Daily Living
CES-D: Centre for Epidemiologic Studies Depression Scale
CFA: Confirmatory Factor Analysis
EFA: Exploratory Factor Analysis
IADL: Instrumental Activities of Daily Living
LASI: Longitudinal Ageing Study in India
NCDs: Non-Communicable Diseases
RMSEA: Root Mean Square Error of Approximation
SDG: Sustainable Development Goal
SEM: Structural Equation Modeling
SRMR: Standardized Root Mean Square Residual
TLI: Tucker-Lewis Index
