# Supplementary material for: Pathways from non-communicable diseases to depression among older adults in India
Source: Soc Psychiatry Psychiatr Epidemiol. 2026 Jun 15;61(12):2545–62. doi: 10.1007/s00127-026-03146-9 (PMC13754998; doi:10.1007/s00127-026-03146-9)
Supplement: Supplementary file 1 [file 127_2026_3146_MOESM1_ESM.docx]

**Table S1. Model Fit Indices for the Structural Equation Modeling Assessing Depression among Older Adults (LASI Wave 1, 2017-18).**

| **Fit Index** | χ² | Degree of Freedom (DoF) | CFI | TLI | RMSEA | SRMR | AIC | BIC |
| --- | --- | --- | --- | --- | --- | --- | --- | --- |
| **Value** | 2055.11 | 9 | 0.923 | 0.82 | 0.062 | 0.035 | 37.93 | 208.75 |

Table S1 presents the overall goodness of fit statistics for the Structural Equation Modeling. The model demonstrated an **acceptable to good fit** to the observed data across multiple indices. The **chi-square (χ²)** value was 2055.11 with 9 degrees of freedom (p< 0.001). Although the chi-square statistic was significant, likely due to the large sample size (n= 59,298), other fit indices indicated that the model adequately represented the underlying data structure.

The **Comparative Fit Index (CFI)** of **0.923** and the **Tucker-Lewis Index (TLI)** of **0.82** suggest that the hypothesized three-factor SEM provided a reasonably close approximation to the observed covariance matrix. Conventionally, CFI values above 0.90 and TLI value i.e, 0.82 indicate an acceptable fit for complex models involving large representative datasets such as LASI. The **Root Mean Square Error of Approximation (RMSEA)** was **0.062**, which falls within the threshold of reasonable approximation error (≤ 0.08), indicating acceptable level of residual variance unexplained by the model. Similarly, the Standardized Root Mean Square Residual (**SRMR)** was **0.035**, well below the 0.05 criterion, reflecting a low average discrepancy between observed and predicted correlations. Information criteria measures, including the **Akaike Information Criterion (AIC= 37.93)** and the **Bayesian Information Criterion (BIC= 208.75)**, were relatively low and indicating optimal parsimony and reduced model complexity without compromising fit.

Overall, the model fit indices collectively support the adequacy of the SEM framework, confirming that the latent structure, comprising **functional limitations**, **lifestyle behaviours** and **NCDs**, provides a theoretically consistent and statistically acceptable representation of the multidimensional determinants of depression among older adults in India and with these acceptable fit indices, the finalized SEM model was retained for interpretation of structural pathways.
